# Real-world experience with gilteritinib maintenance following allogeneic transplantation in relapsed/refractory AML patients harboring *FLT3* mutations

**DOI:** 10.1007/s44313-026-00144-3

**Published:** 2026-06-03

**Authors:** Yun-Tzu Lin, Xavier Cheng-Hong Tsai, Feng-Ming Tien, Yuan-Yeh Kuo, Mei-Hsuan Tseng, Jia-Hau Liu, Bor-Sheng Ko, Chieh-Lung Cheng, Sheng-Chieh Chou, Ming-En Lin, Chien-Chin Lin, Ming-Kai Chuang, Ming Yao, Hsin-An Hou, Wen-Chien Chou, Hwei-Fang Tien

**Affiliations:** 1 Division of Hematology, Department of Internal Medicine, National Taiwan University Hospital, Taipei, Taiwan; 2 Division of Hematological Oncology, Department of Internal Medicine, Chi-Mei Medical Center, Tainan, Taiwan; 3 Tai-Chen Cell Therapy Center, National Taiwan University, Taipei, Taiwan; 4 Department of Hematology and Oncology, National Taiwan University Cancer Center, National Taiwan University Hospital, Taipei, Taiwan; 5 Department of Laboratory Medicine, National Taiwan University Hospital, Taipei, Taiwan; 6 Division of General Medicine, Department of Internal Medicine, National Taiwan University Hospital, Taipei, Taiwan; 7 Department of Internal Medicine, Far-Eastern Memorial Hospital, New Taipei, Taiwan

**Keywords:** Gilteritinib, Post-HSCT maintenance, FMS-like tyrosine kinase 3 (*FLT3)*, *FLT3* inhibitor, Acute myeloid leukemia

## Abstract

**Background:**

Gilteritinib is an established *FLT3* inhibitor used to treat patients with relapsed or refractory (R/R) acute myeloid leukemia (AML) harboring *FLT3* mutations. Prior studies have predominantly evaluated gilteritinib as a posttransplant maintenance therapy in patients who undergo allogeneic hematopoietic stem cell transplantation (allo-HSCT) in complete remission. Nonetheless, evidence remains limited for patients who receive gilteritinib as salvage therapy in a relapsed or refractory setting, proceed to allo-HSCT, and subsequently continue gilteritinib maintenance. This real-world study aimed to address this knowledge gap in an Asian cohort.

**Method and Result:**

Patients with *FLT3*-mutated R/R AML received gilteritinib before transplantation, and those achieving engraftment without grade ≥ 2 acute graft-versus-host disease were offered posttransplant maintenance between days 30 and 90. The cohort (median age, 48 years; 92.0% *FLT3*-ITD) achieved a complete response (CR) rate of 76.2%. At a median follow-up of 46.2 months, gilteritinib maintenance therapy was associated with longer relapse-free and overall survival and a significantly lower 1-year cumulative incidence of relapse. Exploratory measurable residual disease (MRD) subgroup analyses suggested a numerically greater survival difference in patients without flow cytometry-detectable MRD before or after transplantation; however, the findings are limited by the small sample size and lack of *FLT3*-targeted next-generation sequencing-based MRD assessment.

**Conclusion:**

Gilteritinib was generally well-tolerated, with no observed increase in cytomegalovirus (CMV)-related or graft-versus-host complications. This study provides real-world evidence in a clinically relevant scenario and supports the use of gilteritinib maintenance in patients with *FLT3*-mutated AML who undergo transplantation during R/R disease.

**Supplementary Information:**

The online version contains supplementary material available at 10.1007/s44313-026-00144-3.

## Introduction

Acute myeloid leukemia (AML) is a heterogeneous group of hematological malignancies characterized by uncontrolled proliferation and impaired progenitor cell differentiation [1]. The complex leukemogenesis, diverse clinical presentations, and distinct prognoses typically observed in patients with AML are primarily driven by genomic alterations in leukemic cells. FMS-like tyrosine kinase 3 (FLT3) is a receptor tyrosine kinase that regulates the proliferation, differentiation, and apoptosis of hematopoietic cells [2, 3]. *FLT3* internal tandem duplication (*FLT3-*ITD) and tyrosine kinase domain (*FLT3*-TKD) mutations are among the most prevalent genetic alterations in AML and are present in approximately 20–25% and 5–10% of all AML cases, respectively [4, 5]. Several studies have shown that the *FLT3*-ITD mutation is associated with poor prognosis in patients with AML [6, 7]. In response, multiple FLT3 inhibitors have been developed and evaluated across various clinical settings, including frontline therapy [8, 9], treatment of relapsed/refractory (R/R) disease [10–12], and posttransplantation maintenance [13, 14].

Gilteritinib, a potent second-generation type 1 FLT3 inhibitor (FLT3i), has markedly improved the management of patients with *FLT3-*mutated AML. Gilteritinib, which has been approved for the treatment of R/R AML, has shown superior efficacy to conventional salvage chemotherapy, as evidenced by the ADMIRAL and COMMODORE trials that reported improved overall survival (OS) and overall response rates (ORRs) [10, 12]. These findings have positioned gilteritinib as a critical therapeutic option for patients who fail to respond to conventional treatments and as a bridging therapy to allogeneic hematopoietic stem cell transplantation (allo-HSCT) [15], the standard of care for eligible patients with *FLT3*-ITD who are in remission [16]. However, the relapse rate after allo-HSCT is high for patients with *FLT3*-ITD [17]. To address this, gilteritinib has been used as a promising maintenance therapy following allo-HSCT with the aim of reducing relapse rates and improving long-term outcomes. Data from the phase 3 MORPHO trial [13] suggested that gilteritinib maintenance in patients undergoing allo-HSCT in first remission improves relapse-free survival (RFS), with notable benefits in patients with measurable residual disease (MRD) either before or after allo-HSCT.

In recent years, real-world studies have evaluated the therapeutic effects of FLT3 inhibitors administered in the posttransplant setting and have mostly focused on sorafenib or midostaurin [18–20]. However, data on the use of gilteritinib as maintenance therapy after allo-HSCT remain limited, particularly in patients with R/R AML prior to transplantation. Although the MORPHO trial has provided important evidence supporting gilteritinib maintenance in a posttransplant setting, the subgroup analyses revealed regional variability in efficacy, with less pronounced benefits observed in Asian populations, potentially owing to differences in access to FLT3 inhibitors or the timing of transplant [21]. Given the limited data in this context, especially among patients with R/R AML before transplantation, further investigation is warranted. In this study, we aimed to explore the role of gilteritinib as bridge therapy for allo-HSCT and posttransplant maintenance therapy in an Asian cohort.

## Methods

### Study design and population

This prospective observational study was conducted between June 2017 and November 2023 at a medical center in Taiwan (approval numbers: 201406099RIND and 201810058RIND). Patients with R/R AML harboring *FLT3* mutations were eligible for this study. All enrolled patients received gilteritinib as a salvage therapy, followed by allo-HSCT. Gilteritinib was discontinued before initiation of the conditioning regimen. Posttransplantation, gilteritinib maintenance was permitted in patients who demonstrated successful engraftment, which was defined as an absolute neutrophil count of ≥ 500/mm^3^ and platelet count of ≥ 20,000/mm^3^, with no transfusion support, no evidence of grade ≥ 2 acute graft-versus-host disease (GvHD), and who were between 30 and 90 days posttransplantation. The patients were categorized into maintenance and non-maintenance groups based on whether they continued receiving gilteritinib therapy posttransplant. The maintenance dose of gilteritinib varied (40, 80, or 120 mg daily). In patients who maintained sustained complete bone marrow remission with full donor chimerism, discontinuation after two years of maintenance therapy should be considered. Decisions regarding continuation or discontinuation after two years were individualized and made at the discretion of the treating physician in discussions with the patient, taking into account the MRD status, donor chimerism, treatment tolerance, and patient preference. The mean daily dose was calculated and used for comparison. Notably, three patients participated in the ADMIRAL trial [10] and received gilteritinib prior to formal approval by regulatory authorities. Patient demographics, clinical characteristics, disease status, and treatment patterns were collected through a comprehensive review of institutional electronic medical records. Risk stratification was performed using features at diagnosis that were grouped based on the refined Medical Research Council (MRC) criteria [22] and the European LeukemiaNet (ELN) 2022 classification system [23]. Tapering of immunosuppressive therapy (IST) was performed according to the institutional protocols. In the absence of acute GvHD, calcineurin inhibitors were generally tapered beginning approximately one month after transplantation, with the goal of discontinuation before six months whenever clinically feasible, particularly given the R/R setting. *FLT3* mutations were identified via fragment analysis, whereas other myeloid mutations were assessed using the TruSight myeloid sequencing panel and HiSeq platform (Illumina, San Diego, CA, USA) [24]. Library preparation and sequencing were performed according to the manufacturer’s instructions. The details of the variant analysis algorithm used to analyze the diagnostic samples have been described previously [25]. Due to the unavailability of MRD evaluation by next-generation sequencing (NGS), MRD was evaluated by multiparameter flow cytometry and/or quantitative PCR targeting specific genes such as *NPM1* and core binding factors, as previously described [26, 27]. The sensitivity of multiparameter flow cytometry was approximately 10⁻^4^, whereas quantitative PCR assays for gene-specific targets achieved a sensitivity of up to 10⁻^5^. Pre- and post-HSCT MRD were evaluated within 30 days before conditioning and on day +21 after transplantation, respectively.

### Study endpoints

The primary endpoint of the study was RFS, which was defined as the length of time from the initiation of allo-HSCT treatment until disease relapse or death from any cause. The secondary endpoint was OS, defined as the length of time from the initiation of allo-HSCT until death or the last follow-up. Efficacy outcomes, including treatment response and relapse, were assessed according to the ELN 2022 guidelines [23]. Adverse events during gilteritinib treatment were evaluated and graded using the Common Terminology Criteria for Adverse Events (CTCAE) v.5.0. Clinically significant cytomegalovirus (csCMV) infection was defined as the development of CMV disease or the initiation of preemptive anti-CMV therapy [28].

### Statistics

Comparisons between continuous variables were performed using the Mann–Whitney *U* test or Kruskal–Wallis test, whereas differences between categorical variables were assessed using the chi-squared test or Fisher’s exact test. Survival probabilities were estimated using the Kaplan–Meier method, and the log-rank test was used to compare survival curves between groups. Multivariate regression analysis was performed using the Cox proportional hazards model to estimate hazard ratios (HRs) and 95% confidence intervals (CIs). All statistical analyses were performed using the R software (v.4.3.3; https://cran.r-project.org/). Statistical significance was defined as a two-sided *P*-value of < 0.05.

## Results

### Clinical characteristics and molecular alterations of patients receiving gilteritinib

Between June 2017 and November 2023, 42 patients with R/R AML harboring *FLT3* mutations were enrolled in the study. Table 1 summarizes the baseline characteristics, molecular profiles, and treatment outcomes of patients, stratified by post-HSCT maintenance therapy status as follows: no maintenance (*n* = 14), maintenance with MRD positivity (*n* = 5), and maintenance with MRD negativity (*n* = 23). The cohort had a median age of 48 years (range, 10–70 years), with 54.8% female patients. Two patients developed secondary AML from a myelodysplastic syndrome/neoplasm (MDS). The majority (78.6%) of patients had intermediate-risk cytogenetics, and according to the 2022 ELN risk classification, 69.0% were classified as intermediate-risk. The most common molecular alteration at diagnosis was *NPM1* mutation (45.2%), followed by *DNMT3A* and *RUNX1* mutations (14.3%). At enrollment, 11 (26.2%) patients were primarily refractory, having received a median of 2.5 prior lines of therapy. In terms of *FLT3* mutations, 39 (92.0%) patients harbored the *FLT3*-ITD mutation, one patient had both *FLT3-*ITD and *FLT3*-TKD mutations, and one patient had only the *FLT3-*TKD mutation. The allelic ratios of *FLT3*-ITD were uniformly distributed. Notably, five patients acquired *FLT3*-ITD at relapse despite the absence of *FLT3*-ITD at the initial diagnosis. Among the patients with *FLT3* mutations at diagnosis, 31 (70.3%) were given FLT3 inhibitors (primarily midostaurin, 67.6%) as part of frontline therapy, whereas the remaining patients were not because of financial issues. Menin inhibitors were not administered to patients with persistent *NPM1* MRD because these were not available in Taiwan during the study period. The conditioning regimens used were myeloablative (MAC) in 35.7% and reduced-intensity (RIC) in 64.3% of the patients. Donor types included matched siblings (38.1%), haploidentical donors (23.8%), matched unrelated donors (31.0%), and mismatched unrelated donors (7.1%). GvHD prophylaxis primarily involved the use of calcineurin inhibitors and mycophenolate mofetil (69.0%). Among the 26 patients who were alive at the last follow-up, five remained on IST, with a median post-HSCT follow-up of 48.6 months in this subgroup.

**Table 1:** Patient characteristics and outcomes stratified by gilteritinib maintenance status

	Total (*n* = 42)	Without maintenance (*n* = 14)	With maintenance (*n* = 28)	
			Post-HSCT MRD + (*n* = 5)	Post-HSCT MRD-(*n* = 23)
Age median (range)	48 (10–70)	60 (27–70)	51 (45–69)	42 (10–70)
Sex				
Male	19 (45.2%)	6 (42.9%)	2 (40.0%)	11 (47.8%)
Female	23 (54.8%)	8 (57.1%)	3 (60.0%)	12 (52.2%)
Cytogenetic risk				
Favorable	2 (4.8%)	0 (0%)	1 (20.0%)	1 (4.3%)
Intermediate	33 (78.6%)	11 (78.6%)	4 (80.0%)	18 (78.3%)
Adverse	7 (16.7%)	3 (21.4%)	0 (0%)	4 (17.4%)
2022 ELN risk				
Favorable	2 (4.8%)	0 (0.0%)	1 (20.0%)	1 (4.3%)
Intermediate	29 (69.0%)	10 (71.4%)	3 (60.0%)	16 (69.6%)
Adverse	11 (26.2%)	4 (28.6%)	1 (20.0%)	6 (26.1%)
Molecular mutation				
*NPM1*	19 (45.2%)	7 (50.0%)	0 (0%)	12 (52.2%)
*DNMT3A*	6 (14.3%)	3 (21.4%)	0 (0%)	3 (13.0%)
*RUNX1*	6 (14.3%)	3 (21.4%)	0 (0%)	3 (13.0%)
Primary refractory				
Yes	11 (26.2%)	2 (14.3%)	3 (60.0%)	6 (26.1%)
No	31 (73.8%)	12 (85.7%)	2 (40.0%)	17 (73.9%)
Retained *FLT3* at relapse	37 (88.1%)	9 (64.3%)	5 (100.0%)	23 (100.0%)
Acquired *FLT3* at relapse	5 (11.9%)	5 (35.7%)	0 (0.0%)	0 (0.0%)
Previous FLT3i^αβ^				
Midostaurin	25 (67.6%)	6 (66.7%)	4 (80.0%)	15 (65.2%)
Sorafenib	2 (5.4%)	0 (0.0%)	0 (0.0%)	2 (8.7%)
Without previous FLT3i	11 (29.7%)	3 (33.3%)	1 (20.0%)	7 (30.4%)
*FLT3* status				
ITD	39 (92.9%)	12 (92.9%)	5 (100%)	22 (95.7%)
TKD	2 (4.8%)	1 (0.0%)	0 (0.0%)	1 (4.3%)
ITD + TKD	1 (2.4%)	1 (7.1%)	0 (0.0%)	0 (0.0%)
*FLT3*-ITD allelic ratio				
Low	21 (52.5%)	9 (69.2%)	2 (40.0%)	10 (45.5%)
High	19 (47.5%)	4 (30.8%)	3 (60.0%)	12 (54.5%)
Response to pre-HSCT gilteritinib				
CR	32 (76.2%)	7 (50.0%)	4 (80.0%)	21 (91.3%)
PR	5 (11.9%)	2 (14.3%)	1 (20.0%)	2 (8.7%)
PD	5 (11.9%)	5 (35.7%)	0 (0.0%)	0 (0.0%)
Conditioning				
MAC	15 (35.7%)	2 (14.3%)	2 (40.0%)	11 (47.8%)
RIC	27 (64.3%)	12 (85.7%)	3 (60.0%)	12 (52.2%)
Donor type				
MSD	16 (38.1%)	3 (21.4%)	1 (20.0%)	12 (52.2%)
HID	10 (23.8%)	3 (21.4%)	1 (20.0%)	6 (26.1%)
MUD	13 (31.0%)	6 (42.9%)	3 (60.0%)	4 (17.4%)
MMUD	3 (7.1%)	2 (14.3%)	0 (0.0%)	1 (4.3%)
GvHD prophylaxis				
CNIs + MTX	13 (31.0%)	0 (0.0%)	2 (40.0%)	11 (47.8%)
CNIs + MMF	29 (69.0%)	14 (100.0%)	3 (60.0%)	12 (52.2%)
Other strategies	16 (38.1%)	9 (64.3%)	2 (40.0%)	5 (21.7%)
DLI	14 (33.3%)	7 (50.0%)	2 (40.0%)	5 (21.7%)
Other medication^γ^	5 (9.5%)	4 (28.6%)	1 (20.0%)	0 (0%)

*Abbreviations*: *FLT3i* FLT3 inhibitor, *CR* complete remission, *PR* partial remission, *PD* progressive disease, *MAC* myeloablative conditioning, *RIC* reduced intensity conditioning, *MSD* matched sibling donor, *HID* haploidentical donor, *MUD* matched unrelated donor, *MMUD* mismatched unrelated donor, *GvHD* graft-versus-host disease, *CNI* calcineurin inhibitor, *MTX* methotrexate, *MMF* mycophenolate mofetil

^α^Among 37 patients with *FLT3* mutations at diagnosis

^β^One patient received both midostaurin and sorafenib at different time points during treatment

^γ^Four patients received venetoclax-based regimens, and one received oral azacitidine

### Treatment response to gilteritinib maintenance therapy and its impact on prognosis

The CR rate was 76.2%, partial response (PR) rate was 11.9%, with progressive disease occurring in 11.9% of patients. With a median follow-up of 46.2 months (range, 1.8–93.1 months), the OS of the entire cohort was not reached (NR), and the RFS was 62.0 months. The median exposure of post-HSCT gilteritinib maintenance was 21.2 months (range, 0.8–85.5 months). Among the 28 patients who received post-HSCT gilteritinib maintenance, 11 (39.2%) electively discontinued therapy while remaining in complete remission, with a median treatment duration of 23.3 months (range, 6.3–34.4 months) in this subgroup. Among these patients, two subsequently experienced relapse at 14.3 and 38.2 months after discontinuation of gilteritinib, whereas the remaining nine (81.8%) patients remained relapse-free at last follow-up. In the univariate analysis, patients with acquired *FLT3* mutations had significantly worse OS and RFS than those without (NR *vs.* 15.4 months, *P* < 0.001; 62.9 *vs.* 8.8 months, *P* = 0.002). Patients with a history of MDS had worse OS than those without (NR *vs.* 11.0 months, *P* = 0.054). However, a higher *FLT3* allelic ratio (cutoff 0.5), cytogenetic risk based on the refined MRC classification, genomic risk based on the ELN 2022 guidelines, initial hyperleukocytosis, and co-occurring mutations were not significantly associated with clinical outcomes. Notably, primary refractory disease was no longer a poor prognostic factor in patients who underwent allo-HSCT followed by gilteritinib maintenance therapy.

With respect to MRD, 7 (43.8%) patients with detectable pre-HSCT MRD achieved MRD negativity after HSCT. OS and RFS were significantly worse among patients with pre-HSCT MRD compared with those without (OS: 18.1 months *vs.* NR, *P* < 0.001; RFS: 9.7 months *vs.* NR, *P* < 0.001) (Supplementary Fig. 1). Similar findings were observed for post-HSCT MRD findings (OS: 18.1 months *vs.* NR, *P* < 0.001; RFS: 12.1 months *vs.* NR, *P* = 0.002) (Supplementary Fig. 2). Interestingly, patients who were MRD-positive before HSCT but MRD-negative after HSCT had outcomes comparable to those who were MRD-negative before and after HSCT (*P* = 0.317 for OS).

**Fig. 1:**
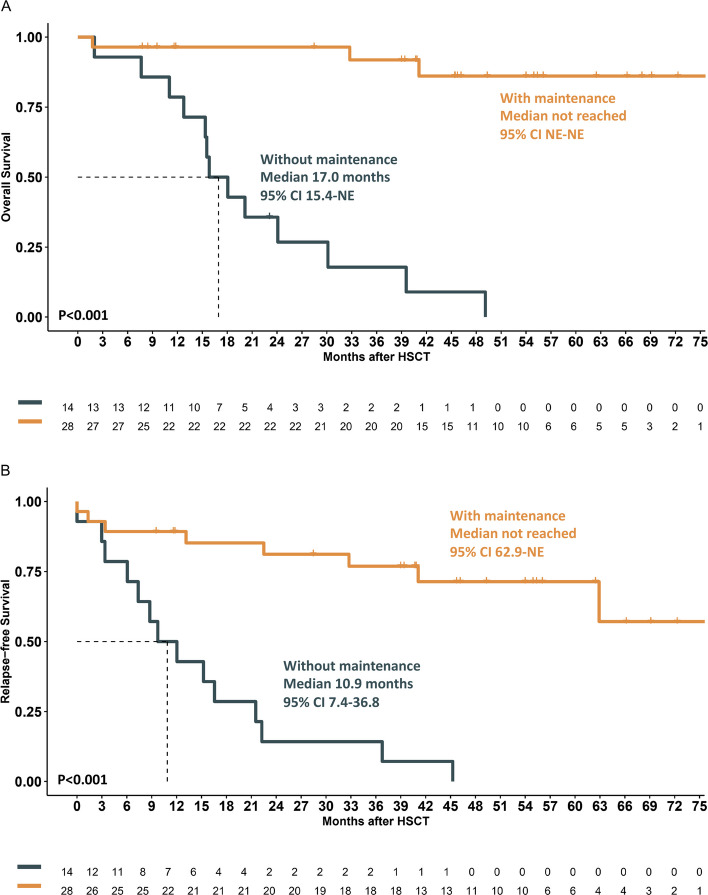
Overall survival (**A**) and relapse-free survival (**B**) of patients who received post-HSCT gilteritinib maintenance or not

The presence of chronic GvHD was also associated with better OS and RFS (OS: NR *vs.* 32.8 months, *P* = 0.008; RFS: NR *vs.* 13.1 months, *P* = 0.002) (Supplementary Fig. 3). Gilteritinib maintenance therapy was a favorable prognostic factor for both OS (NR *vs.* 17.0 months, *P* < 0.001) and RFS (NR *vs.* 10.9 months, *P* < 0.001) (Fig. 1A, B). The 1-year cumulative incidence of relapse (CIR) was significantly lower in the gilteritinib maintenance group (10.7% ± 5.8% *vs.* 14.3% ± 9.4%, *P* < 0.001). Additionally, we included post-HSCT MRD status, presence of chronic GvHD, and gilteritinib maintenance as covariables in the multivariate Cox proportional hazards regression analysis model, which remained independent favorable prognostic factors for both OS and RFS (OS: HR 0.086, *P* = 0.002; RFS: HR 0.148, *P* = 0.002). With respect to HSCT-related complications, the use of gilteritinib did not increase the risk of csCMV infection (*P* > 0.9) and no cases of posttransplant lymphoproliferative disorders were observed. To explore whether the effect of gilteritinib maintenance varied according to MRD status, we performed exploratory subgroup analyses stratified by pre- and post-HSCT MRD status, as assessed by flow cytometry. Among patients with detectable pre-HSCT MRD, no statistically significant differences in OS or RFS were observed between the gilteritinib and non-gilteritinib groups. However, any interpretation of this subgroup is limited by the small sample size and the high frequency of alternative post-transplant interventions in the non-gilteritinib group (Table 1), including venetoclax-based therapy and donor lymphocyte infusion, both of which may have mitigated the relapse risk.

In contrast, among patients with no detectable pre-HSCT MRD, gilteritinib maintenance was associated with significantly improved OS and RFS (OS: NR *vs.* 20.6 months, *P* < 0.001; RFS: NR *vs.* 13.8 months, *P* < 0.001) (Supplementary Fig. 4). Similar patterns were observed when stratified by post-HSCT MRD status (OS: NR *vs.* 22.1 months, *P* < 0.001; RFS: NR *vs.* 13.1 months, *P* < 0.001)(Supplementary Fig. 5). Importantly, these subgroup analyses were not powered to detect treatment–MRD interactions and were based on flow cytometry rather than *FLT3*-targeted NGS MRD, which is now considered a more sensitive and specific modality.

### Adverse events profile

Among patients receiving gilteritinib maintenance, no active GvHD greater than grade 2 was observed, and no patient died from GvHD or GvHD-related complications. Two patients experienced grade II transaminitis, which either improved or stabilized with no dose modification. One patient presented with increased creatine kinase levels despite dose reduction, leading to discontinuation of gilteritinib.

## Discussion

Several studies have shown that allo-HSCT may mitigate the adverse prognostic effects of *FLT3*-ITD in patients with AML [29, 30]. Consistently, our study demonstrated that patients with R/R AML who underwent allo-HSCT had the potential to achieve long-term remission, especially following adequate pre-HSCT disease control and posttransplant maintenance therapy with gilteritinib. Notably, the survival outcomes in our gilteritinib maintenance cohort were comparable to those reported in clinical trials involving patients who achieved CR before transplantation [15]. In this study, 67.6% of the patients reported prior exposure to FLT3 inhibitors before receiving gilteritinib. The favorable outcomes observed in this subgroup support the efficacy of gilteritinib in patients previously treated with FLT3 inhibitors, which is consistent with previous studies [31].

Given the heterogeneity in disease biology and treatment courses inherent to this observational study, we performed subgroup analyses to assess the effects of gilteritinib maintenance in patients stratified by MRD status. Notably, gilteritinib maintenance therapy was associated with significantly improved RFS and OS, particularly in patients who were MRD-negative prior to allo-HSCT. This finding contrasts with the results of the MORPHO trial [13], which reported improved RFS with gilteritinib maintenance, primarily in patients with detectable *FLT3*-ITD MRD before or after HSCT. Conversely, exploratory analyses from the SORMAIN trial [14] suggested that patients with undetectable MRD before allo-HSCT derived the greatest benefits from sorafenib maintenance therapy. Several factors may have accounted for these discrepancies. First, differences in patient selection may have contributed to the inconsistent results. Although the MORPHO and SORMAIN trials enrolled patients in CR, our study included patients with R/R AML. However, as our cohort consisted exclusively of patients who successfully underwent allo-HSCT, it is likely that they had a relatively preserved performance status, which may partially explain the superior outcomes observed. Second, the methods used to assess MRD varied across studies. An ultrasensitive PCR–NGS hybrid assay was employed in the MORPHO trial [13, 32] whereas multiparameter flow cytometry and quantitative PCR (only for *NPM1* mutations) were utilized in both the SORMAIN trial and in our study, which might have lowered the sensitivity [33]. Nonetheless, MRD evaluations in this study were interpreted by experienced hematopathologists using standardized institutional protocols. Third, in our study, some patients in the non-maintenance group received alternative maintenance treatments, including venetoclax-based regimens, which, although not part of a standardized protocol, more closely reflect real-world clinical practice (Table 1).

Previous studies highlighted the poor prognostic implications of concurrent mutations in *FLT3*, *NPM1*, and *DNMT3A* in AML [34, 35]*.* Our data did not reveal significantly inferior outcomes for this subset. This discrepancy may reflect the effect of allo-HSCT and the use of gilteritinib maintenance therapy to mitigate the adverse effects of co-mutations. Additionally, preclinical studies have demonstrated that gilteritinib promotes interleukin-15 production in *FLT3-*ITD–positive leukemic cells, thereby enhancing graft-versus-leukemia effects without actively exacerbating GvHD [36, 37]. Our clinical data support this mechanism, as none of the patients receiving gilteritinib maintenance developed grade ≥ 3 GvHD or died from GvHD-related complications. Importantly, our study demonstrated that gilteritinib maintenance therapy is well tolerated. Only two patients experienced grade II transaminitis, which resolved without dose adjustment, and one patient discontinued gilteritinib owing to creatine kinase elevation. These findings highlight the favorable safety profile of gilteritinib maintenance in a posttransplant setting.

In conclusion, our study provides real-world evidence supporting the efficacy and safety of gilteritinib maintenance therapy following allo-HSCT in patients with R/R *FLT3*-mutated AML. Gilteritinib maintenance therapy was associated with significantly improved RFS and OS rates. Notably, favorable outcomes were observed in patients with prior FLT3 inhibitor exposure, suggesting that gilteritinib may be effective in this population. Prospective studies are warranted to validate these findings and to design optimized maintenance approaches based on molecular and MRD profiles.

## Supplementary Information

Supplementary Material 1.

## Data Availability

The datasets generated during and/or analyzed during the current study are available from the corresponding author on reasonable request.
