# Supplementary material for: Real-world experience with gilteritinib maintenance following allogeneic transplantation in relapsed/refractory AML patients harboring FLT3 mutations
Source: Blood Res. 2026 Jun 3;61(1):26. doi: 10.1007/s44313-026-00144-3 (PMC13234079; doi:10.1007/s44313-026-00144-3)

Real-World Experience with Gilteritinib Maintenance Following Allogeneic Transplantation in Relapsed/Refractory AML Patients Harboring *FLT3* Mutations

Yun-Tzu Lin[1, 2], Xavier Cheng-Hong Tsai[1], Feng-Ming Tien[1], Yuan-Yeh Kuo[3], Mei-Hsuan Tseng[3], Jia-Hau Liu[4], Bor-Sheng Ko[1,3,4], Chieh-Lung Cheng[1], Sheng-Chieh Chou[1], Ming-En Lin[1], Chien-Chin Lin[5], Ming-Kai Chuang[5], Ming Yao[1], Hwei-Fang Tien[1,6], Hsin-An Hou[1,7], Wen-Chien Chou[1]

Supplementary Figure 1. Overall survival (A) and relapse-free survival (B) of patients with or without pre-HSCT MRD.

(A)

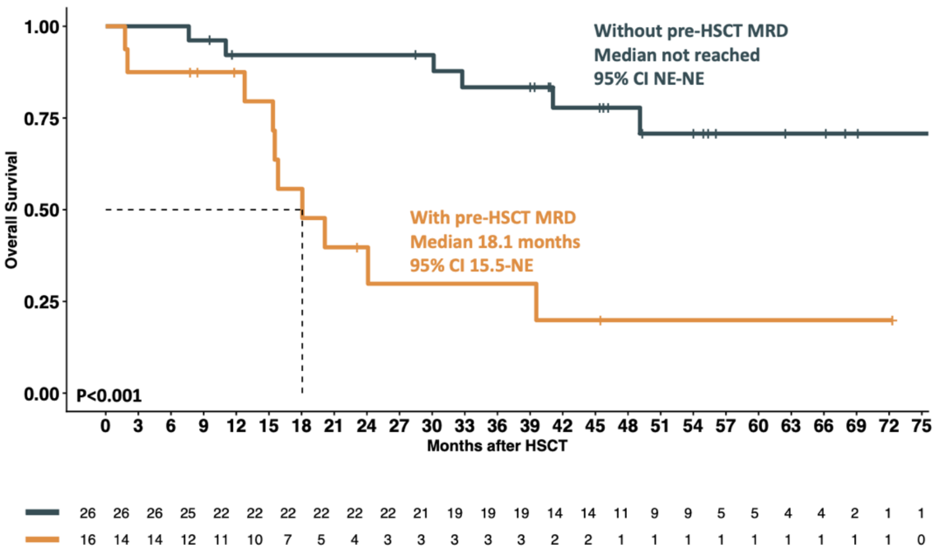

(B)

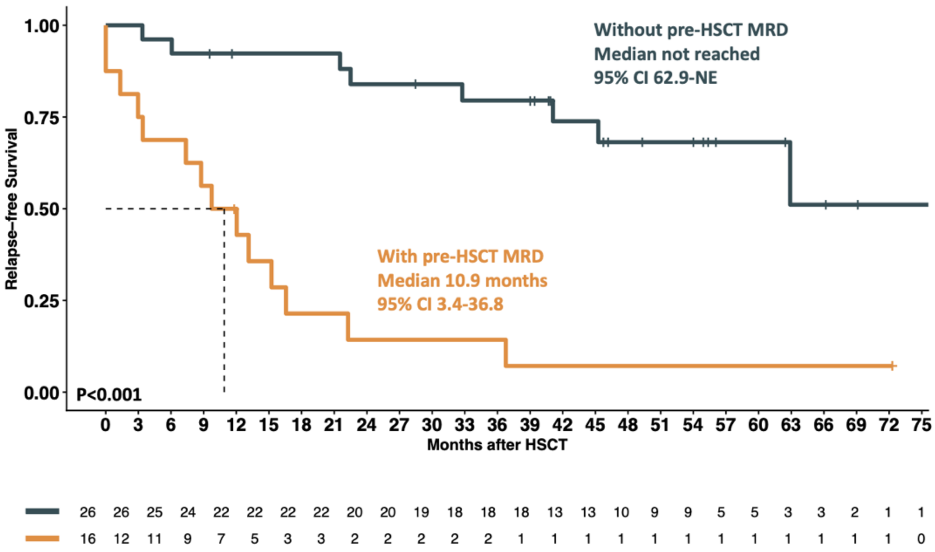

Supplementary Figure 2. Overall survival (A) and relapse-free survival (B) of patients with or without post-HSCT MRD.

(A)

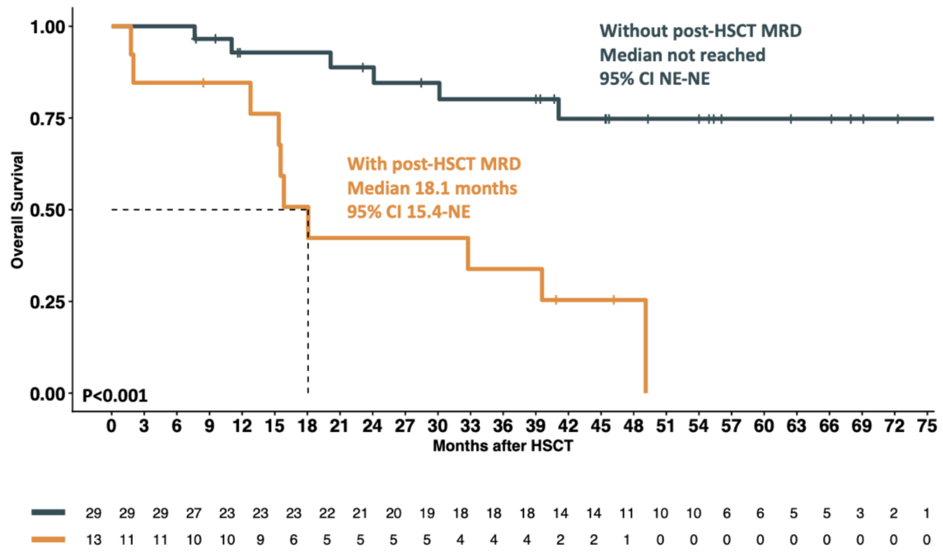

(B)

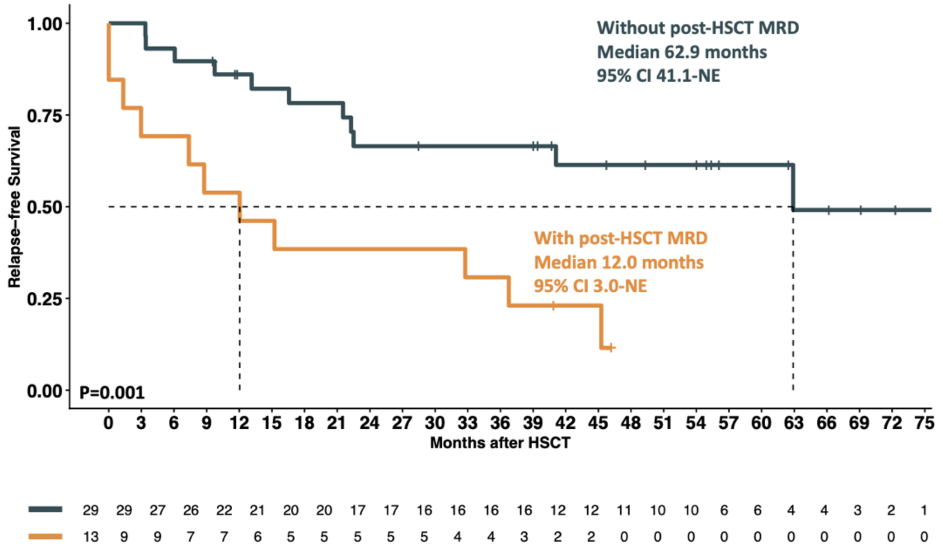

Supplementary Figure 3. Overall survival (A) and relapse-free survival (B) of patients with or without cGvHD.

(A)

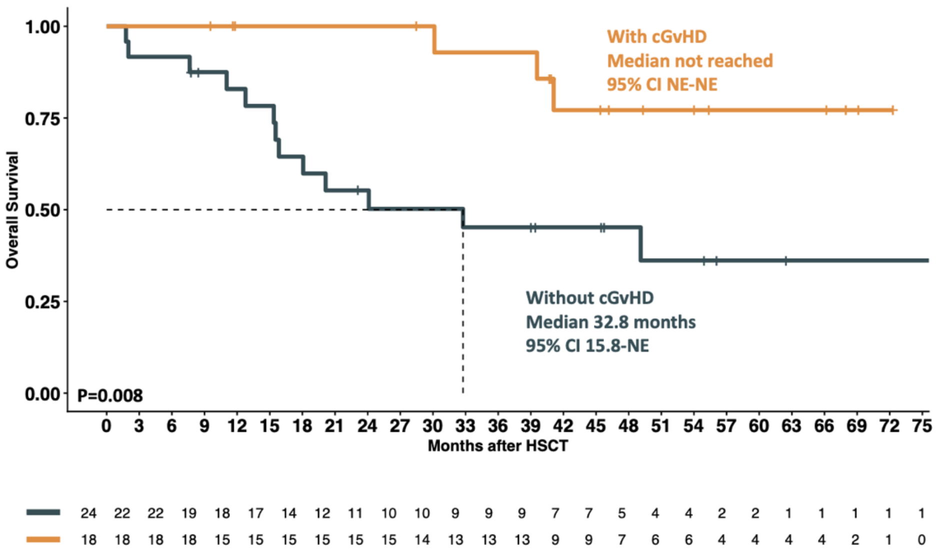

(B)

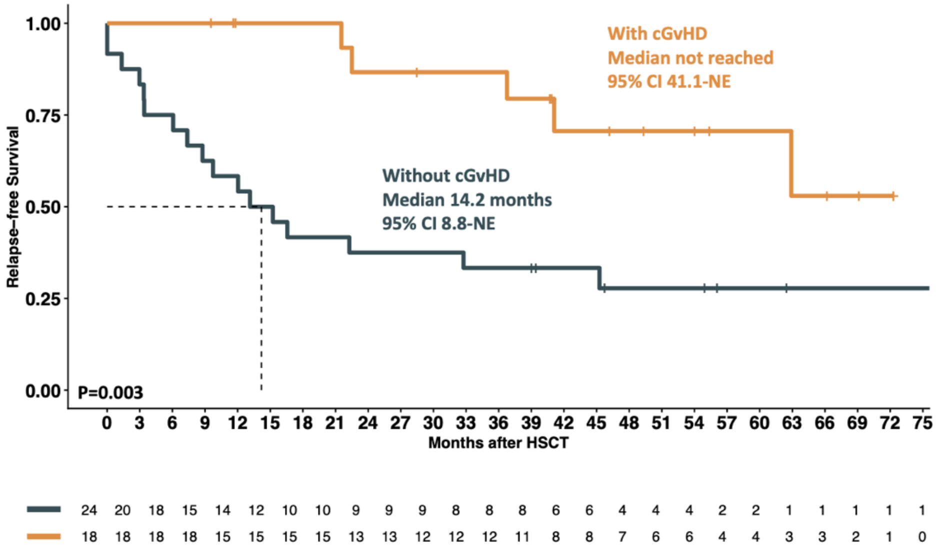

Supplementary Figure 4. Overall survival (A) and relapse-free survival (B) of patients without pre-HSCT MRD who received post-HSCT gilteritinib maintenance or not.

(A)

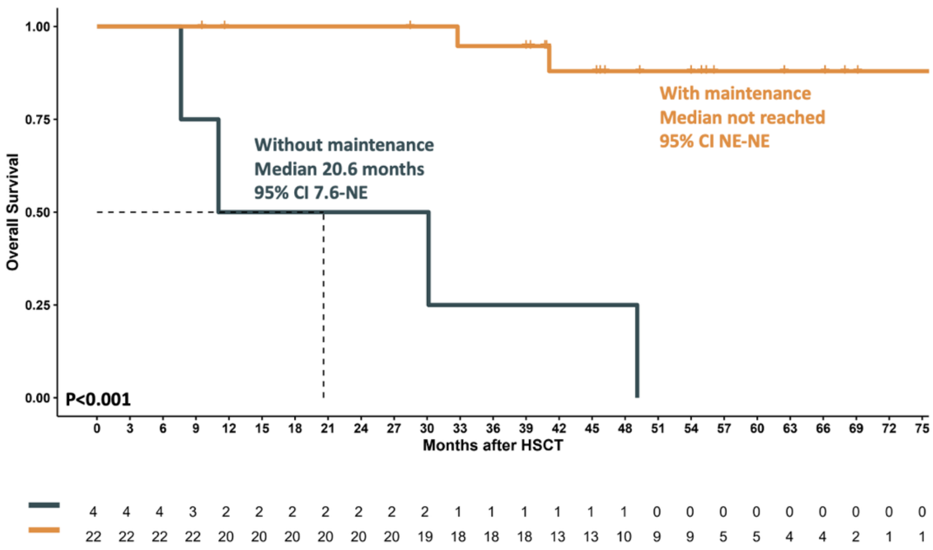

(B)

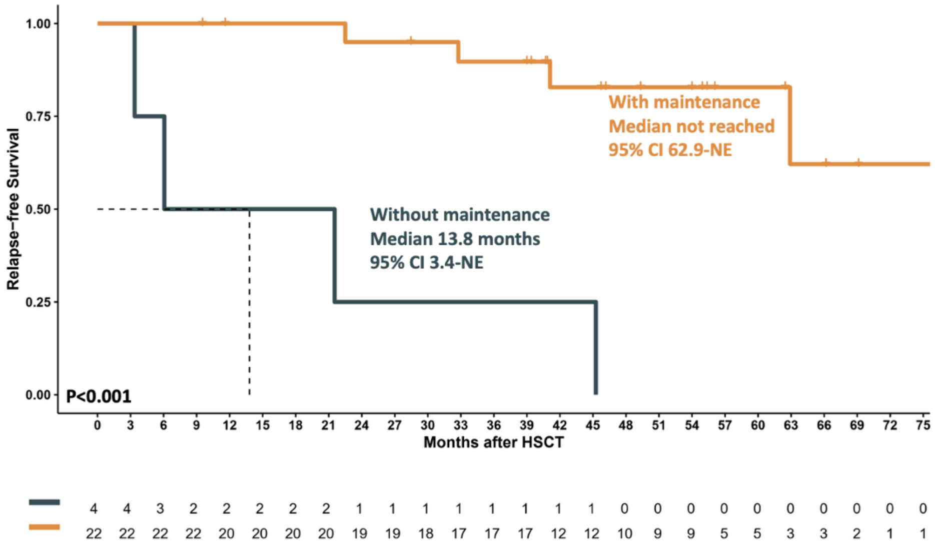

Supplementary Figure 5. Overall survival (A) and relapse-free survival (B) of patients without post-HSCT MRD who received post-HSCT gilteritinib maintenance or not.

(A)

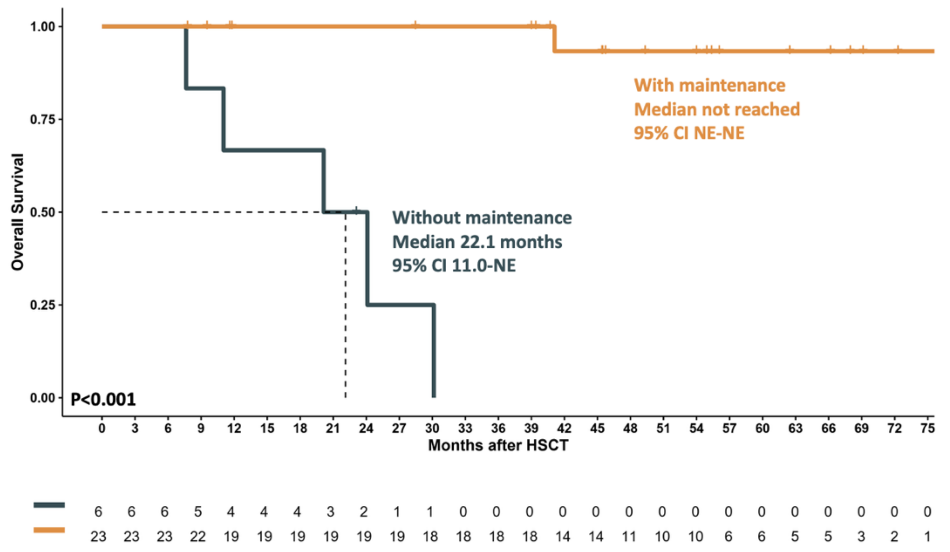

(B)

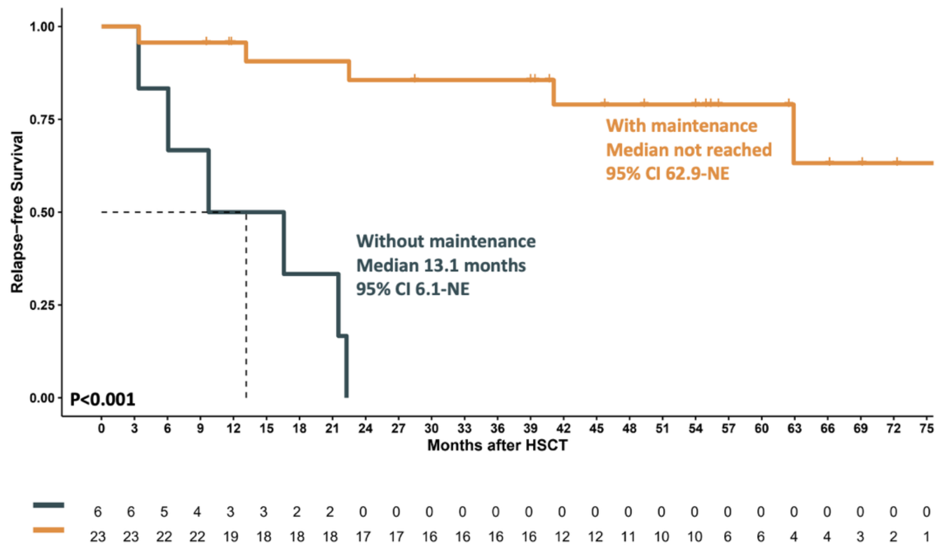

Supplement: Supplementary file 1 — Supplementary Material 1. [file 44313_2026_144_MOESM1_ESM.pdf]
